# Genetic differences in the HLA region contribute to the variability in SARS‐CoV‐2 vaccine responsiveness of older persons: the Doetinchem Cohort Study

**DOI:** 10.1002/cti2.70058

**Published:** 2025-11-05

**Authors:** Yunus Kuijpers, M Liset Rietman, H Susan J Picavet, Peter Engelfriet, W M Monique Verschuren, Anne‐Marie Buisman

**Affiliations:** ^1^ Centre for Prevention, Lifestyle and Health National Institute for Public Health and the Environment (RIVM) Bilthoven The Netherlands; ^2^ Julius Centre for Health Sciences and Primary Care University Medical Centre Utrecht, Utrecht University Utrecht The Netherlands; ^3^ Centre for Immunology of Infectious Diseases and Vaccines National Institute for Public Health and the Environment (RIVM) Bilthoven The Netherlands

**Keywords:** ageing, antibody responses, COVID‐19 vaccination, GWAS, polygenic regulation

## Abstract

**Objectives:**

Older persons generally have weaker antibody responses to vaccines than younger individuals, but heterogeneity is large. We aimed to identify genetic variants associated with primary SARS‐CoV‐2 vaccine‐induced antibody responses in older persons that might contribute to this heterogeneity.

**Methods:**

Demographic and genotype data were collected in the Doetinchem Cohort Study prior to the COVID‐19 pandemic. Antibody responses were measured 1 month after the first and second SARS‐CoV‐2 vaccinations, and genome‐wide association analysis was performed in 842 and 890 individuals respectively. Polygenic scores were calculated and tested in an independent sample, and the variance explained by the scores was estimated using a bootstrap procedure. Genes were mapped to genome‐wide suggestive (*P* < 1 × 10^−5^) loci, and gene set enrichment was performed using the hypergeometric test.

**Results:**

Antibody responses 1 month after the first and second SARS‐CoV‐2 vaccinations were linked to genome‐wide significant (*P* < 5 × 10^−8^) loci on Chromosome 5. Polygenic scores related to these antibody responses could explain 9% (95% CI P1: [−4% to 21%], 95% CI P2: [−4% to 24%]) of the variance. Genome‐wide suggestive loci related to the responses after two vaccinations could be mapped to several genes in the human leukocyte antigen (HLA) region on chromosome 6p21.

**Conclusion:**

Genetic variation is suggested to play a role in the primary vaccine‐induced IgG antibody responses to SARS‐CoV‐2 in older persons. The most prominent source of variation was found to lie in HLA genes, which are enriched in several immune pathways and immune‐mediated diseases.

## Graphical abstract

We identified genetic variants that contribute to the heterogeneity in primary SARS‐CoV‐2 vaccine‐induced antibody responses in older persons. Most prominently were genetic variants in the HLA region that were associated with lower anti‐SARS‐CoV‐2‐S1 IgG antibody responses and could be linked to various immune pathways and immune‐mediated diseases.

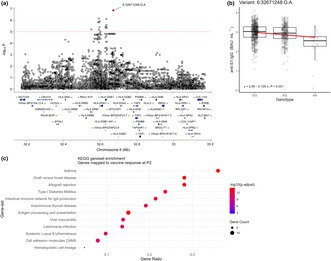

## Background

The COVID‐19 pandemic has had devastating global consequences and disproportionately affected older persons.^1^ Older persons were at higher risk of severe illness and mortality, which is probably mostly because of the age‐related decline in immune system functioning known as immunosenescence.^2^ This decline impairs the ability to generate effective immune responses against new pathogens such as SARS‐CoV‐2.

Especially in older persons, vaccination remains the most effective strategy to lessen COVID‐19's impact and mortality.^3^ However, there are considerable differences in vaccine‐induced antibody responses among individuals. This heterogeneity in antibody responses upon vaccination against SARS‐CoV‐2 has been shown to be especially high in older persons.^4^ In addition, factors such as sex, underlying health conditions and genetic makeup also contribute to this heterogeneity in immune responses and disease manifestation.^5^, ^6^ Environmental factors such as prior exposure to SARS‐CoV‐2 and lifestyle factors have also been shown to affect vaccine‐induced immune responses.^7^

Besides environmental and physiological factors, genetics also contribute to the heterogeneity both in the health status of older persons and in immune functioning.^8^ Genome‐wide association studies (GWAS) have been used to study the influence of genetic variation on a multitude of diseases and complex traits.^9^ Identified associations have also been linked to specific regions in the genome that provide potential functional explanations, some of which involve their impact on immune functioning. Particularly, the human leukocyte antigen (HLA) region on Chromosome 6 has been associated with vaccine responses after hepatitis B vaccinations^10^ and breakthrough infections after COVID‐19 vaccinations.^11^

In our study, we aimed to analyse the association of the genetic background with primary vaccine‐induced SARS‐CoV‐2 IgG antibody responses in older individuals aged 50 to 92 by using the Doetinchem Cohort Study (DCS).^12^, ^13^ By conducting a GWAS, we identified genetic variants associated with IgG antibody responses both 1 month after the first and after the second vaccine dose of the primary vaccination series with mRNA BNT162b2 (Pfizer) or AZD1222 (Astra Zeneca) in infection‐naïve individuals. In addition, by using gene set enrichment analysis (GSEA), we aimed to identify potential immune‐mediated pathways that may partly account for the heterogeneity in antibody responses upon vaccination of older persons. Overall, this study enhances our understanding of the relationship between genetics and primary vaccine‐induced antibody responses in an ageing population.

## Results

### Study population

**Figure 1:**
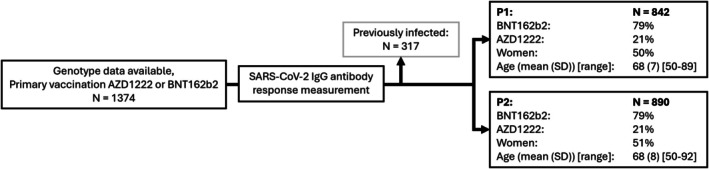
Flow chart of the Doetinchem Cohort Study participants with genotype data selected for those of the SARS‐CoV‐2 vaccine study subgroup. At 1 month after the first primary SARS‐CoV‐2 vaccination (P1) 842 participants were included and 1 month after the second vaccination (P2) 890 participants were included. Demographics of the study population are shown per timepoint.

From a total of 1374 participants, who all had genotype data available, SARS‐CoV‐2 IgG antibody responses were determined post a primary vaccination with either AZD1222 or mRNA BNT162b2. This resulted in 842 and 890 individuals who were infection‐naïve and had antibody concentrations measured 1 month after their first vaccination dose (P1) and second dose (P2), respectively Of these participants, 21% received a primary vaccination with AZD1222 and 79% received a primary vaccination with BNT162b2. Sex, age and vaccine type were similarly distributed across both time points (Figure 1).

### Loci on Chromosome 5 are associated with SARS‐CoV‐2 IgG antibody responses after both a first and second vaccine dose

**Figure 2:**
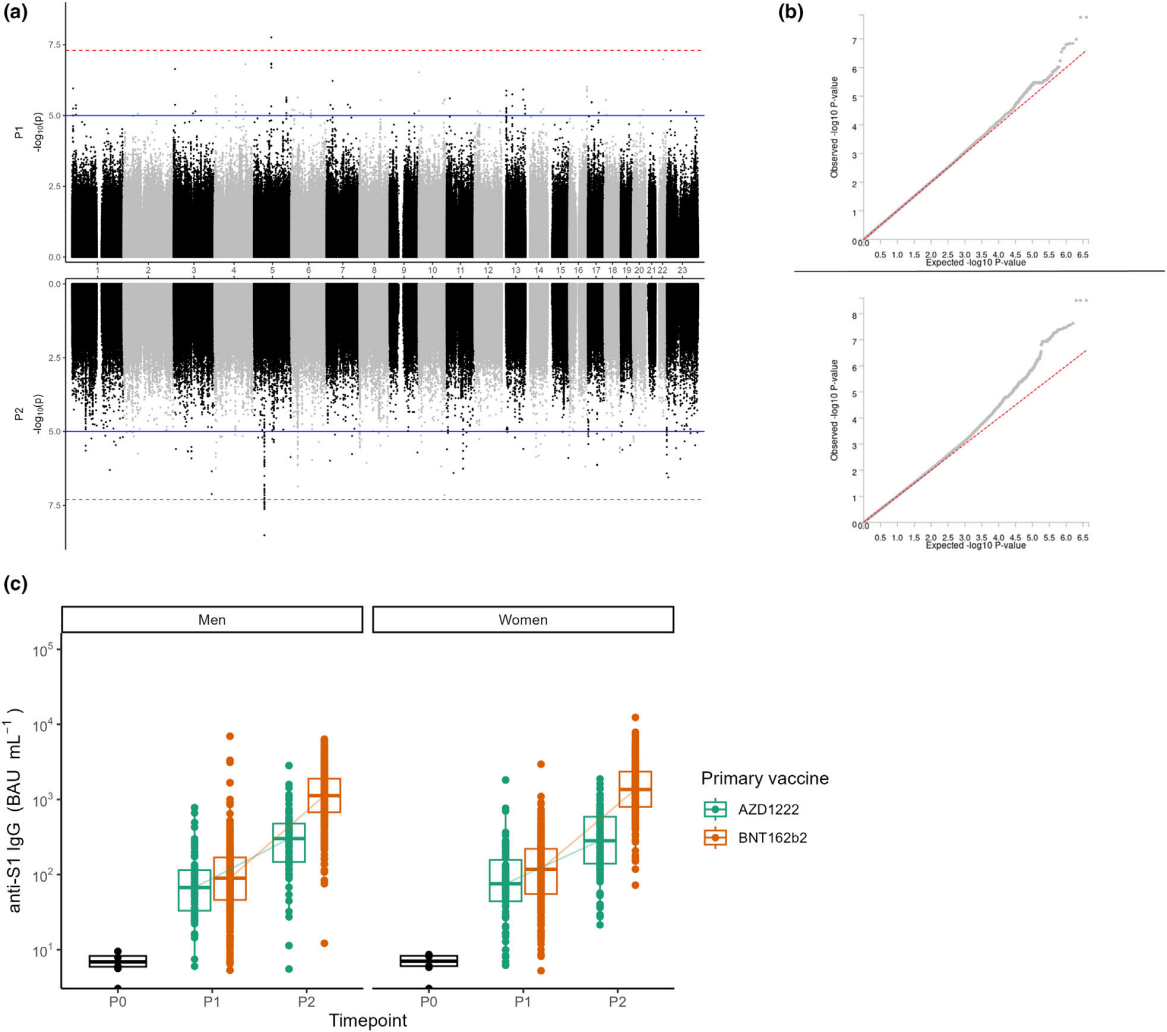
Genome‐wide association study (GWAS) results with vaccine‐induced SARS‐CoV‐2‐S1 IgG antibody responses 1 month after a first primary (P1) or a second SARS‐CoV‐2 vaccine dose (P2), corrected for vaccine type. **(a)** Miami plot of genome‐wide associations following the first (P1, upper) and second vaccination (P2, lower). The blue line indicates genome‐wide suggestive associations (*P* ≤ 1e−5) and the dotted red line indicates genome‐wide significant associations (*P* ≤ 5e−8). **(b)** Q‐Q plot of genome‐wide associations following the first (P1, upper) and second vaccination (P2, lower). The red diagonal line represents the predicted associations and grey dots represent the observed associations. **(c)** Anti‐SARS‐CoV‐2‐S1 IgG antibody responses before vaccination (P0), and 1 month after the first (P1) and second (P2) vaccine dose, separated by sex and vaccine type (AZD1222 in green and mRNA BNT162b2 in red).

Testing for associations between genetic variants and antibody responses showed two independent loci on Chromosome 5 that were genome‐wide significantly associated with the antibody responses 1 month after a first or second vaccine dose (Figure 2a and b). Additionally, the overall antibody responses within both men and women used for this analysis at 1 month after the first dose (P1) and the second dose (P2) with either AZD1222 or mRNA BNT162b2 increased on average compared to pre‐vaccination (P0). However, antibody concentrations showed a large heterogeneity within the entire study population (Figure 2c). The C allele of locus 5:85135387:T:C was associated with a lower antibody concentration 1 month after the first vaccination (Beta: −1.63, *P* < 1.75 × 10^−8^) (Table 1) and the T allele of locus 5:51642657:C:T with a lower antibody concentration 1 month after the second vaccination (Beta: −0.23, *P* < 3.07 × 10^−9^) (Table 2). *P*‐values for associations with additional loci on Chromosomes 3, 4, 6, 10, 11, 22 and the X‐chromosome were almost genome‐wide significant (*P* ≤ 5 × 10^−7^) for antibody responses after a first or second dose, as can be seen in Tables 1 and 2, respectively. All of these variants were located in the intergenic region. Full summary statistics for all genome‐wide associations 1 month after a first and second vaccination are provided in Supplementary tables 1 and 2, respectively.

**Table 1:** The top independent genome‐wide significant (*P* ≤ 5 × 10^−8^) and nearly significant (*P* ≤ 5 × 10^−7^) associations with the SARS‐CoV‐2 IgG antibody responses 1 month after a first SARS‐CoV‐2 vaccination

ID	Chromosome	Position	*P*‐value	Effect allele	Other allele	Beta	SE
5:85135387:T:C	5	85 135 387	1.75 × 10^−8^	C	T	−1.63	0.29
22:37546292:G:A	22	37 546 292	1.03 × 10^−7^	A	G	−2.09	0.39
4:151245237:G:A	4	151 245 237	1.52 × 10^−7^	A	G	−1.18	0.22
3:7610154:G:A	3	7 610 154	2.26 × 10^−7^	A	G	−1.15	0.22
10:1922714:G:T	10	1 922 714	2.87 × 10^−7^	T	G	−2.27	0.44

**Table 2:** The top independent genome‐wide significant (*P* ≤ 5 × 10^−8^) and nearly significant (*P* ≤ 5 × 10^−7^) associations with the SARS‐CoV‐2 IgG antibody responses 1 month after a second SARS‐CoV‐2 vaccination

ID	Chromosome	Position	*P*‐value	Effect Allele	Other Allele	Beta	SE
5:51642657:C:T	5	51 642 657	3.07 × 10^−9^	T	C	−0.23	0.04
10:125839419:C:T	10	125 839 419	7.07 × 10^−8^	T	C	−0.49	0.09
3:185227707:G:T	3	185 227 707	7.65 × 10^−8^	T	G	−0.5	0.09
6:32671248:G:A	6	32 671 248	1.38 × 10^−7^	A	G	−0.13	0.02
X:10076870:C:T	X	10 076 870	2.81 × 10^−7^	T	C	−0.33	0.06
11:33978671:T:C	11	33 978 671	3.99 × 10^−7^	C	T	−0.32	0.06

### Nine percent of the variance in SARS‐CoV‐2 induced IgG antibody responses was explained by genetic variation

**Figure 3:**
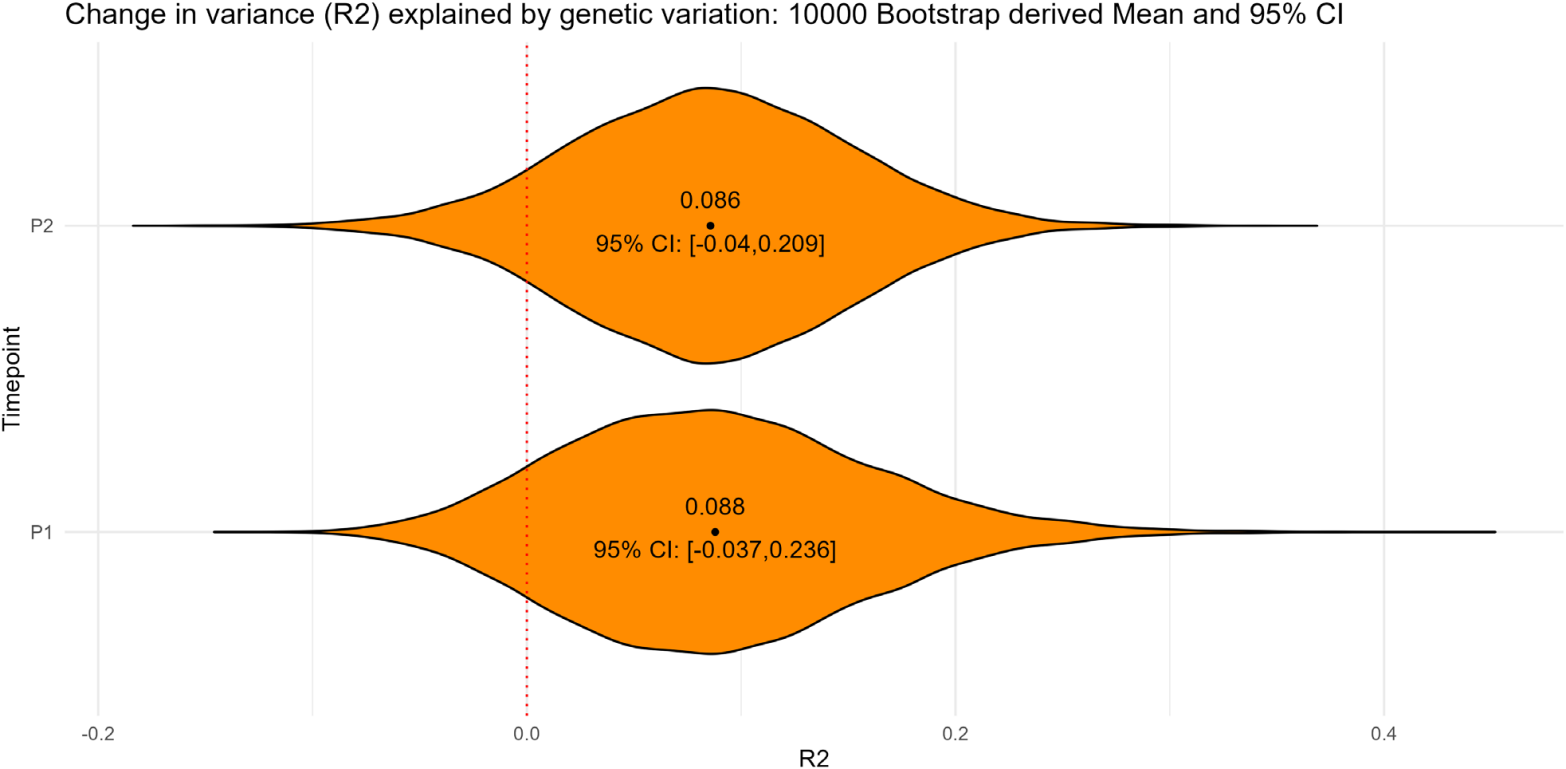
Variance (R2) in SARS‐CoV‐2 S1 IgG antibody responses explained by polygenic scores 1 month after a first primary (P1) and second (P2) vaccine dose. Model estimates and confidence intervals were derived using a 10 000‐fold bootstrap procedure.

In order to assess to what extent genetic factors contributed to the SARS‐CoV‐2 IgG antibody responses in this older population, we constructed polygenic scores to model the antibody responses. Using a bootstrap procedure, we estimated that on average close to 9% of the variance in antibody responses, both at P1 and P2, could be explained by genetic variation (Figure 3). However, the 95% confidence interval included 0 (−0.04 to 0.209 for P1, −0.037 to 0.236 for P2).

### Gene set enrichment reveals immune‐mediated pathways especially related to HLA genes associated with the SARS‐CoV‐2 IgG antibody responses after vaccination

**Figure 4:**
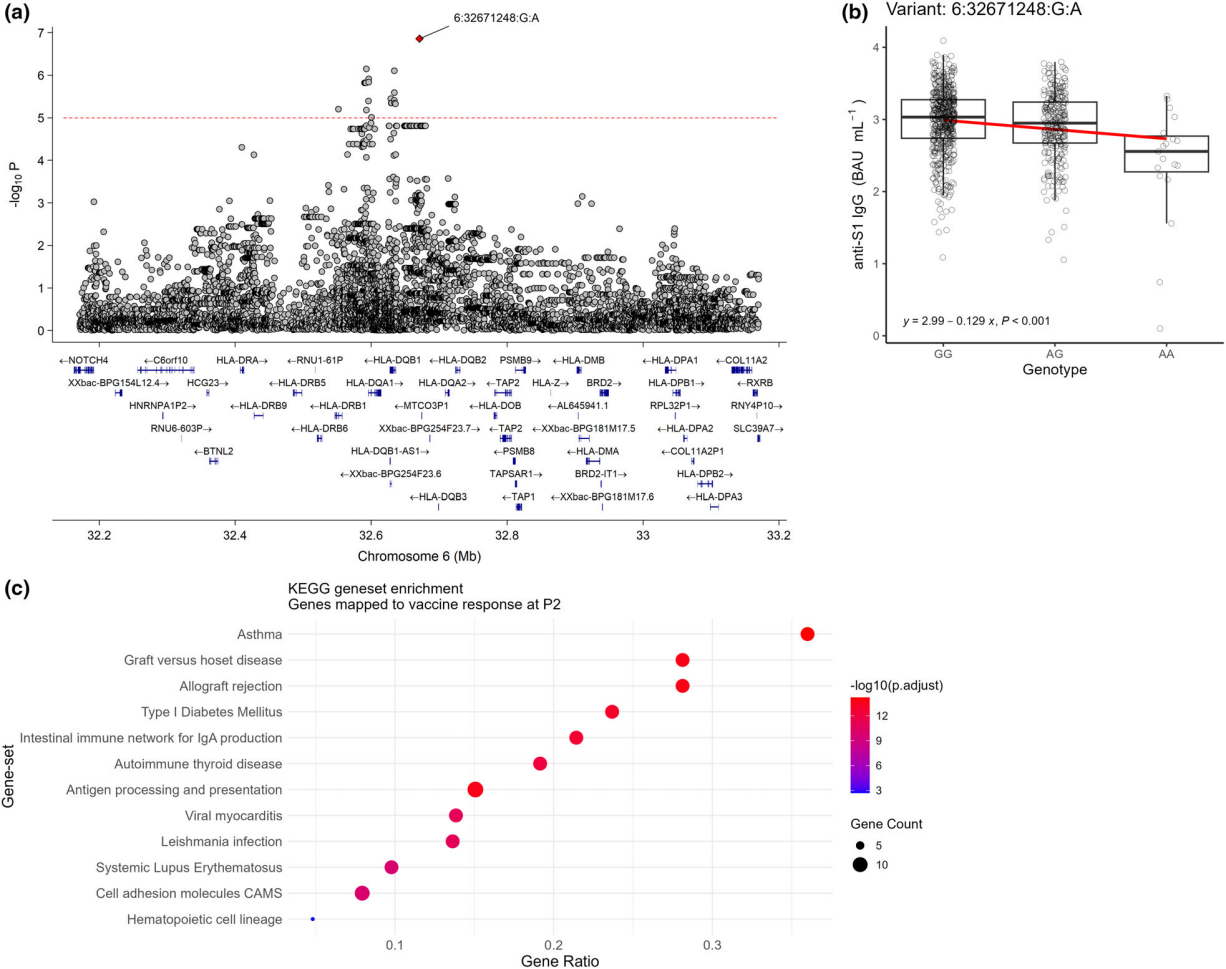
**(a)** Locus‐Zoom plot of the HLA region on Chromosome 6 for GWAS associations with the SARS‐CoV‐2‐S1 IgG vaccine responses at 1 month after the second primary vaccination (P2). A dotted red line is shown at the genome‐wide suggestive threshold (*P* ≤ 1e−5) used for identifying associations to be included in gene set enrichment. **(b)** Anti‐SARS‐CoV‐2‐S1 IgG antibody concentrations at P2 separated by genotype dosage for variant 6:32671248:G:A. **(c)** Dot plot showing KEGG gene set enrichment. Genes included were positionally mapped to genome‐wide suggestive loci associated with the antibody responses at P2. Dot size indicates the number of genes overlapping with the gene set. Position on the x‐axis indicates the ratio of overlapping genes versus the total gene set. Dot colour indicates the level of significance after multiple testing correction using the Bonferroni procedure.

Gene set enrichment using the KEGG pathway database was performed after positionally mapping genes to all genome‐wide suggestive (*P* ≤ 1 × 10^−5^) loci associated with antibody responses after vaccination. Mapped genes and their respective GWAS variants are displayed in Supplementary tables 3 (*N* = 1082) and 4 (*N* = 1906) for responses 1 month after a first and second vaccine dose, respectively. No enriched gene sets could be identified using the genes that were associated with antibody responses at P1. However, at P2, many HLA genes such as HLA‐DRB1 and HLA‐DQB1 were mapped to genome‐wide suggestive loci on Chromosome 6 (Figure 4a). In addition, we observed differences in the antibody concentrations of our study population at P2 based on the effect allele of variant 6:32671248:G:A, the lead SNP of interest on Chromosome 6 (Figure 4b). The effect allele (A) was associated with increasingly lower antibody concentrations. The identified HLA genes, among other genes mapped to loci at P2, were enriched in gene sets linked to (dys)functioning of immune responses, such as seen in autoinflammatory diseases or in immunity to infectious diseases, or to specific aspects of (immune) cell functioning (Figure 4c).

## Discussion

This study aimed to explore possible genetic factors influencing the antibody response to SARS‐CoV‐2 vaccination in older COVID‐19 infection‐naïve persons. Through a GWAS, we identified two significant loci on Chromosome 5 that are associated with antibody responses both measured 1 month after the first and after the second vaccine doses. The overall results of our study suggest that specific genetic variants contribute to differences in antibody responses to a (new) primary vaccine in the older (> 50 years) population. Furthermore, several genome‐wide suggestive loci were mapped to the HLA region on chromosome 6p21, known for its role in immune function. Gene set enrichment analysis indicated that these loci are involved in immune‐related pathways and diseases. Polygenic score analysis estimated that these genetic loci could explain about 9% of the variability in antibody responses, although this estimate was not statistically significant, possibly because of the limited sample size.

The estimated 9% variance explained by genetic factors is lower than what similar studies on vaccine response heritability have reported. For instance, research conducted in various twin studies has shown that heritability in relation to vaccine responsiveness can range between 36% in a cohort consisting of adults aged 18 and 65 years and 77% in children 5 months of age.^14^ This, however, varies greatly depending on the vaccine type and age of the vaccines. It has been shown previously that older individuals have, on average, a weaker but more heterogeneous antibody response in comparison with younger adults.^15^ This heterogeneity could indicate that in an older population the influence of genetic factors on the variability in antibody response upon vaccination has become less than in a younger population. This is likely because of the multifactorial nature of effects on the functioning of the immune system, with major contributions from non‐genetic exposome influences, such as comorbidities, which are likely to increase with age.^6^

Other research on the genetic association with COVID‐19 vaccine responses has also highlighted the significance of the HLA genomic region, showing its (modest) role in antibody response variability.^11^, ^16^ The HLA region is crucial for presenting antigens to T cells, thereby influencing the immune response. Variations in HLA genes can affect how effectively the immune system recognises and responds to vaccines or pathogens including SARS‐CoV‐2.^17^ Downstream, these helper T‐cell responses help to shape B‐cell immunity and antibody response. In the context of COVID‐19, certain HLA alleles have been associated with differences in disease susceptibility and severity.^18^ Our findings support the idea that genetic factors, especially those involving the HLA region, are important in shaping individual responses to COVID‐19 vaccination in older persons. However, we identified different relevant SNPs associated with both the first and second primary vaccine dose. This could potentially mean that some genes in the HLA region are involved in the naïve immune responses at P1 and others in the memory immune responses at P2. Previously, genetic variation has been linked to differences in surface antigen recognition and presentation as well as the expression of specifically HLA‐DRB1 and HLA‐DQB1.^10^, ^11^ This in turn affected the hepatitis B vaccine response,^10^ which is in accordance with our findings regarding the same locus, and the response to COVID‐19 breakthrough infections.^11^ It is noteworthy that a superantigen motif has been identified on the spike protein that strongly binds to T‐cell receptors and forms a ternary complex with MHCII molecules.^19^, ^20^ The consequences of weaker vaccine responsiveness are particularly relevant for older adults who are prone to experience reduced vaccine effectiveness. Weaker vaccine responses have been observed in older persons for anti‐influenza and pneumococcal vaccines.^21^, ^22^

Other genes such as RXRB, which lie within the extended MHC region but outside the classical HLA region, were also identified in association with the SARS‐CoV‐2 antibody responses. This indicates that there is a possibility for other genes outside the HLA genes to play a relevant role in influencing the vaccine‐induced primary antibody response. In addition, other studies have also identified that certain cytokine gene variants^23^ and complement signalling transcriptional signatures^24^ influenced antibody concentrations after vaccination against COVID‐19. This highlights the importance of genetic factors outside the HLA region for influencing antibody responsiveness. Gene set enrichment analysis was performed with the goal of identifying potential biological processes or phenotypes that could further explain the differences in antibody responses after vaccination or could be targets for future research. While the genetic variants that had the strongest associations with the antibody response were located on Chromosome 5, this region did not lead to the identification of any specific gene sets. This suggests that these variants affect the processes underlying the antibody response only indirectly or that our study lacked the power to properly identify the relevant processes. We did however identify multiple immune‐mediated diseases as well as pathways such as antigen processing and presentation by using genes located within the HLA region. This might lead to further identification of potential risk groups of older adults with a genetic predisposition related to low immune responses to primary vaccination.

A key strength of this study is the use of the well‐established DCS, which is a sample of the Dutch general population. This allowed us to study the antibody responses to a primary vaccine in an ageing stratum of the general population, where most vaccination studies include older persons from a clinical care setting. Thanks to the comprehensive data collected during the vaccination study, we were also able to accurately exclude previously infected persons from our analyses. This allowed us to better explore the role of genetic variation in antibody responses to a primary vaccine in an aged population. However, the relatively small sample size for the GWAS may have limited our ability to detect additional significant associations, as well as the power to accurately estimate the amount of explained variance with polygenic scores. Because of this smaller sample size, our study did not have enough power to draw strong conclusions on the non‐HLA genes we identified. Therefore, we were not able to stratify our genetic analyses based on vaccine type, age or gender and could only correct for these variables. Possible differences between these groups should be studied in more detail to identify potentially different mechanisms in men and women, or in younger and older persons as a consequence of immunosenescence. Given that older persons on average have weaker but more heterogeneous antibody responses after vaccination with BNT162b2,^15^ possibly as a consequence of immunosenescence, it could mean that with more power additional genetic variants could be identified related to immunosenescence that indirectly influence antibody responses. The lack of an independent replication cohort for this GWAS, and the lack of previously published GWAS studies in a comparable population currently limits the generalisability of our findings. Additionally, our study population was ethnically homogeneous, which, while reducing certain confounding factors, may limit the applicability of our results to more diverse groups.

This study provides evidence that genetic factors, particularly residing within the HLA region, contribute to the variability in antibody responses following SARS‐CoV‐2 vaccination in an older population. Although the estimated variance explained by genetic factors was not statistically significant, likely because of the small sample size, our findings suggest that genetics contributes to variability in vaccine‐induced antibody responses in older persons. Further research with larger populations is needed to validate these findings and to better understand the mechanisms by which genetic variation influences vaccine‐induced immunity.

## Methods

### Study population

We used the longitudinal population‐based DCS that started in 1987 including individuals 20–59 years of age who were re‐examined every 5 years.^12^, ^13^ Participants of the DCS were eligible for the GWAS if they had completed the primary vaccination series of either 2 AZD1222 vaccine doses or 2 mRNA BNT162b2 vaccine doses. This resulted in our study population of ageing and older adults with an age range from 50 to 92 years of age (*n* = 1374). Infection status was assessed based on self‐reported test results or by seropositivity for SARS‐CoV‐2 IgG antibodies against spike protein or nucleoprotein (at P0, prior to vaccination), or against only nucleoprotein (P1, P2). Persons infected pre‐vaccination were excluded from the analyses in this study, and samples were labelled as infection‐naïve if they were uninfected up to the time point for which they were used. The 842 infection‐naïve participants 1 month after the first vaccination (P1) and 890 infection‐naïve participants after the second vaccination (P2) all had genotype data available. Note that there were more participants at P2 as some persons could not be included before P1 because of a lack of P1 sample.

### Genotyping, quality control and imputation

Genotyping, quality control and imputation were performed as described previously.^25^ Genomic DNA was isolated from venous blood samples and genotyped in the HUman GEnomics Facility (HUGE‐F) (Rotterdam, the Netherlands) using the Illumina Infinium Global Screening Array‐24 Kit (GSA).^26^

The R package GenABEL 1.8–0^27^ was used to perform initial quality control for both participants and genetic variants. Participants were excluded if there was a sex mismatch (*n* = 45), samples were duplicates (*n* = 18) or were the second of a pair of monozygotic twins (*n* = 1), heterozygosity rate was high [false discovery rate (FDR) < 1%] (*n* = 37), the sample call rate was <0.95 (*n* = 20), or if they were ethnic outliers based on visual inspection of a kinship matrix made using genomic principal component analysis (*n* = 114). Variants were excluded if they were monomorphic (*n* = 109 129), if they had a low genotype call rate (< 95%) (*n* = 8005), were out of Hardy–Weinberg equilibrium (FDR < 0.2) (*n* = 0), and X‐linked markers if they were likely to be autosomal (*n* = 421). Subsequently, the HRC‐1000G‐check‐bim.pl script from Rayner (http://www.well.ox.ac.uk/~wrayner/toolsHRC‐1000G‐check‐bim.plscript) was used for further quality control and to convert the Plink genotype data^28^ to separate VCF files per chromosome. This pre‐imputation step of quality control filtered/excluded additional SNPs based on genotype call rate < 10^−6^ (*n* = 0).

Finally, genotypes were imputed to the Haplotype Reference Consortium (HRC) panel (version r1.1 2016)^29^ with the Michigan Imputation Server^30^ using NCBI Genome Reference Consortium Human Build 37. Pre‐phasing was performed on the imputation server with Eagle v2.3^29^ and imputation with Minimac3.^31^

### Sample collection post SARS‐CoV‐2 vaccination

Blood samples and questionnaires were taken at 1 month post first (P1) and second (P2) SARS‐CoV‐2 vaccination (EudraCT 2021‐001976‐40 and NL74843.041.20, EudraCT 2020‐003620‐16). Questionnaires covered demographic factors, vaccination background and COVID‐19 infection status based on self‐testing or testing at a laboratory. Finger‐prick blood (NL76551.041.21) was self‐collected in microtubes and returned by mail. In addition, blood samples obtained by vena puncture were collected (NL76719.041.21, EudraCT 2021‐002363‐22). Serum was isolated from each sample by centrifugation and stored at −80°C until sample measurement.

### SARS‐CoV‐2 IgG antibody response measurement

Immunoglobulin G (IgG) antibody responses against Spike (S1) and Nucleoprotein (N) were measured simultaneously using a bead‐based multiplex assay as previously described.^32^ IgG concentrations were calibrated against the International Standard for human anti‐SARS‐CoV‐2 immunoglobulin (20/136 NIBSC standard) and expressed as binding antibody units per millilitre (BAU mL^−1^). The threshold for seropositivity was set at 10.1 BAU mL^−1^ for Spike S1^33^ pre‐vaccination and 14.3 BAU mL^−1^ for Nucleoprotein^34^ post vaccination.

### Genome‐wide association study

The associations between SNPs and anti‐S1 SARS‐CoV‐2 IgG antibody responses 1 month after a first and second vaccine dose were tested using a generalised linear model per SNP, using Plink 2.0.^28^ To reduce confounding and account for potential population stratification, we corrected for age, sex, vaccine type and the first three principal components (PCs) derived from a principal component analysis on the genetic variation within the population. In addition to quality control as described above, prior to analysis, variants with a call rate lower than 0.05, a minor allele frequency smaller than 0.01, and those with a Hardy–Weinberg *P*‐value smaller than 10^−6^ were filtered out. Genome‐wide significance was defined using a *P*‐value threshold of *P* ≤ 5.0 × 10^−8^ and a genome‐wide ‘suggestive threshold’ of *P* ≤ 1.0^−5^ was used for functional follow‐up analyses.

### Polygenic scoring

To derive polygenic scores, genome‐wide association was assessed in a random subset of 80% of our cohort. The derived scores were validated in the remaining 20% of the study participants using PLINK 2.0. Polygenic scores were calculated for a range of GWAS *P*‐value thresholds (from *P* ≤ 0.5 to *P* ≤ 10^−5^). We selected the most statistically significant variants within windows of 250 k of base pairs, excluding all variants with a linkage‐disequilibrium (LD) score of 0.1 or higher. We then multiplied the dosage of the remaining alleles by the effect sizes of the variants in the GWAS performed in the 80% subset. By summing these scores across all independent variants passing our *P*‐value thresholds, we calculated a polygenic score for each person per threshold. We then constructed linear models for 10% of our samples to estimate the variance in antibody responses explained by the polygenic scores at the various *P*‐value thresholds. This was done to select a single threshold that best explained the variance. This resulted in a final linear model using a GWAS *P*‐value threshold of *P* ≤ 10^−5^.

#### Functional analysis

We used all genome‐wide suggestive variants identified with the GWAS performed in our entire cohort to map these to genes and perform a pathway enrichment analysis. Independent variants were selected within a 100‐k distance in base pairs from one another and positional gene mapping using 500‐kb windows was performed. Subsequently, the GENE2FUNC pipeline of the Functional Mapping and Annotation (FUMA) for GWAS platform^35^ was applied to test for enrichment of the identified genes in the KEGG database.^36^ We used the Benjamini‐Hochberg (FDR) method to account for multiple testing.

## Author contributions

**Yunus Kuijpers:** Conceptualization; data curation; formal analysis; visualization; writing – original draft; writing – review and editing. **M Liset Rietman:** Conceptualization; data curation; supervision; writing – original draft; writing – review and editing. **H Susan J Picavet:** Conceptualization; supervision; writing – original draft; writing – review and editing. **Peter Engelfriet:** Conceptualization; supervision; writing – original draft; writing – review and editing. **W M Monique Verschuren:** Conceptualization; project administration; supervision; writing – original draft; writing – review and editing. **Anne‐Marie Buisman:** Conceptualization; project administration; supervision; writing – original draft; writing – review and editing.

## Conflict of interest

The authors declare no conflict of interest.

## Ethics statement

The study was conducted according to the principles of the World Medical Association Declaration of Helsinki and its amendments since 1964, and in accordance with the Medical Research Involving Human Subject Act (WMO). The study protocols were approved by the Medical Ethics Committee of the University Medical Center Utrecht and all informed consent was obtained from all participants. Ethics approval was for finger‐prick blood sampling in the majority of the DCS participants (NL76551.041.21, EudraCT 2021‐001976‐40) and for finger‐prick blood sampling as well as venapunction in a small part of the participants (NL74843.041.20, EudraCT 2020‐003620‐16 and NL76719.041.21, EudraCT 2021‐002363‐22).

## Supporting information


Supplementary table 1

Supplementary table 2

Supplementary table 3

Supplementary table 4

## Data Availability

We welcome collaboration. For use of the available data, please contact Professor W. M. M. Verschuren or H. S. J. Picavet (doetinchemstudie [at] rivm [dot] nl).
